# Cross-cultural measurement invariance of the multidimensional competitive orientation inventory: Non-clinical samples in South Korea and Hungary

**DOI:** 10.1371/journal.pone.0337685

**Published:** 2025-12-26

**Authors:** Taeyoon Aum, Márta Fülöp, Seoyoung Kim, Jeongeun Diana Lee, Dong-gwi Lee

**Affiliations:** 1 Department of Psychology, Yonsei University, Seoul, Republic of Korea; 2 HUN-REN Institute of Cognitive Neuroscience and Psychology, Research Centre for Natural Sciences, Budapest, Hungary; 3 Károli Gáspár University of the Reformed Church in Hungary, Budapest, Hungary; 4 Yonsei Psychological Science Innovation Institute, Yonsei University, Seoul, Republic of Korea; Public Library of Science, UNITED STATES OF AMERICA

## Abstract

The Multidimensional Competitive Orientation Inventory (MCOI) aims to assess competitive orientations including self-developmental, hypercompetitive, avoidant, and indifferent attitudes. The present study tested its cross-cultural applicability and examined cross-national differences between South Korea (*n* = 350) and Hungary (*n* = 343), taking cultural traits into account. Using a revised 10-item version (two low-loading items removed), multi-group CFA with WLSMV supported configural and scalar invariance across countries. Factor variances were invariant, whereas factor covariances were not, indicating that inter-factor correlations differed across countries despite a shared structure. For criterion validity analyses, we conducted cross-group comparisons only for measures that demonstrated invariance; noninvariant measures were interpreted within each country. Overall, the results suggest that the MCOI can be used cross-culturally with appropriate scale adjustment. However, competitive orientations may manifest differently across individualistic and collectivistic cultures in both the motivations for and consequences of competition, underscoring the need for further research. Implications for the practical use of the MCOI and directions for future research are discussed.

## Introduction

Competition refers to a social situation in which individuals participating in an activity are awarded unequally based on relative performance [1]. In these situations, competitive orientation describes various personal attitudes toward competition [2]. Prior to the 1990s, studies on competitive orientation [3,4] tended to focus on an excessive desire to win, which was deemed the opposite of cooperativeness. Therefore, competitive orientation was viewed as a unidimensional concept [5], a negative attitude that can lower individuals’ creativity, personal and organizational productivity, and the quality of their relationships [6,7]. However, this view has changed with the emergence of a non-binary approach suggested by Deutsch [8], arguing that conflict is a mixture of competitive and cooperative processes, rather than being mutually exclusive [5]. The non-binary approach extends the idea that people engage in conflicts through mixtures of attitudes, which can lead to both adaptive and maladaptive consequences [9]. In the 1990s, competitiveness was studied less in relation to cooperation, and researchers interested in adaptive aspects of competitive attitudes started to focus more on differentiating types of competitiveness (e.g., [10–13]). These studies showed that different competitive orientations are related to distinct intrapersonal and interpersonal characteristics, supporting the conception that competitive orientations are multifaceted.

Ryckman et al. [11–13] developed measures for “hypercompetitive attitude,” “personal-development competitive attitude” and “competition avoidance,” based on Horney’s [14] psychodynamic theory to capture the maladaptive/neurotic and adaptive competitive orientations, but with three discrete tests. The measures could show the levels of adaptiveness and maladaptiveness but did not define the multidimensional factor structure. As a result, comparisons or discussions about different dimensions of competitive orientations were limited. Several other multidimensional measures followed: “Competitiveness/Mastery Questionnaire” [10], “Dislike of Competitive Situations” [15], and “Competitive Orientation Measure” [16]. Still, some attributes of competitive orientations remained unassessed in these multidimensional scales. For one, indifferent attitudes toward competition, previously thought to indicate a lack of competitive orientation [17], may have distinct psychological characteristics that are valuable for understanding competitiveness. Additionally, some of the scales (e.g., [11–13]) assessed either approaching or avoiding attitudes toward competition; however, no scales assessed both aspects at once. These limitations indicated the need for a scale that embraces a comprehensive spectrum of attitudes toward competition including approaching, avoiding, and indifferent orientations.

To address these limitations, Orosz et al. [17] developed and validated the Multidimensional Competitive Orientation Inventory (MCOI) with Hungarian samples. The MCOI was developed through a phenomenological approach based on an open-ended survey administered to people from diverse nationalities and occupations to explore personal attitudes toward competition. The MCOI has been validated primarily using Exploratory Structural Equation Modeling (ESEM), Confirmatory Factor Analysis (CFA), and convergent validity analysis. It also has been validated with a wide range of participants, including adolescents from elementary through high school, and adults up to the age of 93. The MCOI measures four factors using twelve items: hypercompetitive orientation, self-developmental competitive orientation, anxiety-driven competition avoidance, and lack of interest in competition. The MCOI can assess not only the approaching and avoidant motivations for competition but also the lack of interest or indifference towards competition and whether one wins or loses.

Approaching competitive motivation includes hypercompetitive and self-developmental competitive orientations. Hypercompetitive orientation is characterized by an excessive and potentially maladaptive desire to win, and may be associated with negative psychological characteristics, such as neuroticism [11,17]. By contrast, a self-developmental competitive orientation involves an interest in personal growth through participation in competition. This orientation can be related to positive psychological characteristics, such as resilience and positivity [12,17]. Conversely, within avoidant motivation, anxiety-driven competition avoidance is conceptually similar to Ryckman’s [13] competition avoidance. However, this orientation stems from anxiety related to the process of competition (one’s experience during competitive activities), while Ryckman [13] explained the avoidance as stemming from one’s concern over losing others’ affection and acknowledgement when failing to win in competitions. Anxiety-driven competition avoidance tends to be associated with negative psychological characteristics, such as perceiving a large discrepancy between the ideal and actual selves [17]. In contrast, lack of interest reflects a neutral attitude toward winning or losing; that is, neither approaching nor avoidant motivations are involved. While validating the MCOI, Orosz et al. [17] delineated the relationships among the four competitive orientations with the following four psychological characteristics: resilience, perfectionism, achievement motivation, and positivity. These have been frequently referred to in the competition literature (e.g., [17–19]).

The MCOI measures the dimensions of competitive orientation using a small number of items. Therefore, it has practical value for studies on diverse social situations involving competition. However, some empirical evidence [20,21] has shown that the MCOI needs cross-cultural validation to ensure its generalizability. In addition, according to Bronfenbrenner’s [22] ecological systems theory, a construct or concept can be understood differently by individuals depending on the sociocultural context. Cultural differences may be related to different factor structures or relationships with other variables such as personal traits and the psychological outcomes of holding a particular competitive orientation.

Prior findings [20,21,23,24] support the necessity of the cross-cultural validation for the MCOI. Qualitative comparisons between Hungarian, Japanese, and Canadian participants [20,21] revealed meaningful cultural differences in their views on competitors, the purpose and process of competition, and their general attitudes toward competitive activities. While Hungarians and Canadians typically perceived competitors as adversaries and associated the purpose of competition with victory and survival, Japanese participants held a distinctive perspective. They viewed competitors as catalysts for mutual improvement and regarded competition as a means of societal growth. In doing so, they rejected a dichotomous (“beauty and the beast,” [21]) view of competition and cooperation.

To clarify these differences, Fülöp [20,21] explored the distinctions between individualistic and collectivistic cultures. The collectivistic Japanese culture tends to discourage individuals from standing out or causing interpersonal tension, thereby fostering an interdependent self-concept [25,26]. Conversely, the individualistic Hungarian and Canadian cultures, which emphasize self-enhancement, may have cultivated an independent self-concept, leading the participants to perceive competition as an opportunity to demonstrate superior abilities [25,26]. Given that cultural characteristics can contribute to the formation of different perceptions, experiences, and uses of one’s competitive orientation, cross-cultural validation of the MCOI with a representative non-Western sample living in a collectivistic country is worthy of study. Furthermore, the cultural differences shown in Fülöp’s qualitative studies [20,21] could be re-examined quantitatively. Recently, Wang et al. [27] confirmed the presence of the competitive orientations proposed by Orosz et al. [17] with Chinese participants; however, the results showed that the relationships with other psychological variables, such as resilience, may vary from those observed in Western samples. Nonetheless, the differences have not yet been directly compared across countries.

South Koreans can be a suitable and interesting sample for studying the competitive orientation because of their collectivistic yet highly competitive characteristics. South Koreans uphold collectivistic values, such as humility [28], similar to the Japanese participants in the qualitative cross-cultural study by Fülöp [20,21], as well as a marked preference for winning, which has been interpreted as an individualistic tendency [29]. For example, the UN Committee on the Rights of the Child stated that South Koreans’ excessive academic competition for entrance into top universities is concerning [30,31]. When Korean adult employees had a higher level of competition-oriented attitude, they also worked harder and were more productive than usual; this relationship between competitiveness and performance was stronger for Koreans than for Chinese and Japanese [23]. Moreover, the seventh World Values Survey [32] showed that South Koreans not only actively participate in competitive activities but also endorse the competition-oriented attitude. The survey results showed that 64.8% of South Korean participants responded that income should differ according to the amount of effort a person puts into their work, compared to 27.6% for Japan and 39.3% for China.

Meanwhile, the collectivistic South Korean culture could engender emotional ambivalence due to the face-saving culture, which emphasizes maintaining a positive social reputation [33]. As one of the display rules to conserve a positive social atmosphere, the direct expression of certain interpersonal emotions, such as jealousy, is often discouraged [26]. This cultural trait contrasts with that of individualistic cultures, which encourage the expression of ego-focused emotions [26,34]. For South Koreans, nonconformity to norms could lead to feelings of inadequacy and social maladjustment [26,29]. The emphasis on harmony may inhibit the formation and expression of strong competitive emotions and attitudes.

In summary, South Korea has a highly competitive social atmosphere while emphasizing socially harmonious behavior. The dual and perhaps somewhat contradictory pressures can elevate stress among South Koreans. For instance, academic perfectionism along with perfectionistic self-presentation to meet social norms could result in psychological struggles, such as anxiety, workaholism, and obsessive-compulsive behaviors [29,35]. Here, to understand the competitive orientation in the South Korean context, this study tested whether the competitive orientation construct could be better explained by a factor structure different from that of the original MCOI, and could have different relationships with psychological characteristics compared to those found in individualistic cultures, such as Hungary (see [36]). In order to verify the cross-cultural applicability of the MCOI, this study aimed to answer the following research questions:

(1) Does the factor structure of the MCOI remain invariant across South Korean and Hungarian samples?

To address this question, a cross-national analysis of measurement invariance was conducted to examine whether the configuration of and relationships among the factors of the scale would remain consistent across different countries.

(2) Do the relationships between competitive orientations and psychological characteristics differ between South Korean and Hungarian cultures?

Since the relationship between a construct and its criterion variables may vary across different samples and their respective cultures (e.g., [27,37]), this study incorporated perfectionism, achievement motivation, positivity, and resilience as the criterion-related variables that are known to be associated with the competitive orientations [17,38].

This study contributes to the competition literature by providing evidence supporting the cross-cultural applicability of the MCOI across cultures. By quantitatively examining cultural differences that have largely been explored through qualitative approaches (e.g., [20,21]), it further extends our understanding of competition across individualistic and collectivistic contexts.

## Methods

### Participants

This study included two samples: one from Korea and the other from Hungary. The Hungarian sample consisted of 343 participants aged 13–59 years (*M* = 19.48, *SD* = 5.09). Other demographic information included gender (female = 214) and education: 54.8% had primary education, 24.8% had secondary education, 19.2% completed college/university-level education, and 1.2% did not specify. The Korean sample consisted of 350 participants aged 14–57 years (*M* = 19.39, *SD* = 5.08). Corresponding demographic information for the Korean sample included gender (female = 219) and education: 59.43% had primary education, 34.86% had secondary education, and 5.71% had completed college/university-level education.

In Chen [39]’s Monte Carlo simulations, unequal group sizes tended to attenuate changes in fit indices, thereby reducing the power to detect noninvariance. In addition, smaller samples were more prone to inflated Type I error rates. Based on these findings, we collected the Korean sample (*n* = 350) that was comparable in size to the existing Hungarian dataset (*n* = 343) [17], resulting in a total of 693 participants for analysis.

### Sampling procedure and data collection

The Hungarian data were obtained from Orosz et al. [17], specifically from their Study 4. Data collection for the Hungarian sample took place between February 15 and July 15, 2014. The study received ethical approval from the Central Research Ethics Committee of the affiliated institution. According to the institution’s regulations, parents of students under the age of 18 provided passive, opt-out consent, as the study was conducted anonymously and did not collect any identifiable data. Informed consent was obtained from participants via the online questionnaire. After reading information about the questionnaire, they were asked whether they wished to proceed (“Yes/No”) before accessing the main survey.

For validation and cross-cultural comparisons, Korean participants with demographic characteristics similar to those of the Hungarian sample were recruited. Data collection for the Korean sample took place from October 25, 2022, to November 3, 2022. The study was approved by the institutional review board (IRB) of the university to which the authors are affiliated. Adult participants provided consent by reading the study description on the online survey page and indicating their agreement via a checkbox. For adolescent participants under the age of 18, written consent was obtained via electronic signatures from both the participant and their legal guardian. Participant recruitment and administration of the anonymous survey were conducted online through Dataspring (https://ko.d8aspring.com/). Reward points convertible to cash were provided by Dataspring as compensation for completing the survey. A total of 350 participants’ responses were retained after excluding 19 cases exhibiting careless responses (e.g., providing the same response across items and measures). The raw data from the South Korean sample has been made available as Supporting Information S1 Data.

### Measures

#### Multidimensional competitive orientation inventory.

The MCOI [17] includes 12 items and four factors: hypercompetitive orientation, self-developmental competitive orientation, anxiety-driven competition avoidance, and lack of interest in competition. Items are rated on a 6-point Likert-type scale ranging from 1 (strongly disagree) to 6 (strongly agree), with a higher score indicating a greater level of competitive attitude in the corresponding dimension. We used Korean translations of the scale developed through a process of translation, back-translation, and cross-linguistic review. First, the original English version was translated into Korean [Lee, Unpublished data]. The translated version was then blindly back-translated into English by independent bilingual reviewers. Native English speakers compared the original and back-translated versions to identify any discrepancies in meaning, and items with questionable equivalence were revised in consultation with the translators. In addition, a reviewer fluent in both Korean and Hungarian examined the Korean–Hungarian compatibility of the translated items.

In the Korean sample, the Cronbach’s alpha and McDonald’s omega were.75/.77 for self-developmental competitive orientation,.87/.87 for hypercompetitive orientation,.63/.68 for competition avoidance, and.42/.55 for lack of interest. Upon removal of one item with a low factor loading from both the competition avoidance and lack of interest, the revised coefficients improved to.71/.71 and.74/.74, respectively. In the Hungarian sample, Cronbach’s alpha and McDonald’s omega values were.87/.87,.87/.87,.87/.87, and.87/.87 for the original items, respectively, and after removing the same items, the coefficients were.86/.86 for competition avoidance and.81/.81 for lack of interest.

#### Connor-Davidson Resilience scale.

The Korean Connor–Davidson Resilience Scale (K-CD-RISC), translated and validated by Baek et al. [40], was used to assess participants’ perceptions of their resilience. The original Connor-Davidson Resilience Scale (CD-RISC) [41] contains 25 items. Orosz et al. [17] used a 10-item version of the CD-RISC [42]. To maintain consistency, this study also focused on the 10 corresponding items from the K-CD-RISC. Each item is rated on a 4-point Likert-type scale ranging from 0 (not true at all) to 4 (true nearly all the time). Higher scores reflect higher degrees of resilience. The Cronbach’s alpha and McDonald’s omega of this scale were.87/.87 in the Korean sample and.87/.87 in the Hungarian sample.

#### Almost perfect scale-revised.

The Almost Perfect Scale-Revised (APS-R) [43] measures perfectionistic tendencies. This study used the Korean version of the Almost Perfect Scale (K-APS-R) translated and validated by Park [44]. The K-APS-R includes 19 items in three dimensions: High Standards (5 items), Order (4 items), and Discrepancy (10 items). The “Order” subscale has been excluded in several studies including Orosz et al. [17], as it is suggested to be inappropriate for distinguishing adaptive and maladaptive tendencies of perfectionism [45]. Accordingly, we used the 15 items that measure high standards and discrepancy. Items are rated on a 5-point Likert scale ranging from 1 (strongly disagree) to 5 (strongly agree). Higher scores indicate higher levels of perfectionistic tendency in the corresponding dimension. To analyze the Hungarian sample under the same item conditions as the Korean sample, four items were removed from the APS-R before conducting the analysis. In the Korean sample, the Cronbach’s alpha and McDonald’s omega values were.78/.79 for high standards and.91/.92 for discrepancy. In the Hungarian sample, these values were.82/.84 and.93/.93, respectively.

#### Consequences of perfectionism.

The Consequences of Perfectionism Scale (COPS) [46] consists of 10 items and measures the positive (6 items) and negative (4 items) consequences of perfectionism. Participants rate each question on a 5-point Likert scale ranging from 1 (extremely untrue of me) to 5 (extremely true of me). Higher scores on each dimension reflect greater levels of either positive or negative consequences of perfectionism. Here, we used a Korean translation of the scale developed through a process of translation, back-translation, and revision based on reviews from native Korean graduate students, and bilingual students fluent in Korean and English. Initially, two graduate students translated the COPS into Korean. The translated version was then blindly back-translated by a bilingual graduate student. Two native English speakers compared the original English and back-translated versions. Questionable items (i.e., items rated below four) were then identified and modified. This process was repeated until the meanings of the translated items were mutually agreed to be equivalent and unambiguous. In the Korean sample, the Cronbach’s alpha and McDonald’s omega values were.87/.87 for positive consequences and.82/.82 for negative consequences. In the Hungarian sample, these values were.89/.89 and.88/.88, respectively.

#### Positivity scale.

The Positivity Scale (POS) [47] assesses positive orientation based on optimism, life satisfaction, and self-esteem. The scale contains 8 items rated on a 5-point Likert scale ranging from 1 (strongly disagree) to 5 (strongly agree). Higher scores indicate higher levels of positivity. We used a Korean translation of the scale developed through the same process as that used for COPS. The Cronbach’s alpha and McDonald’s omega values were.86/.86 for the Korean sample and.83/.84 for the Hungarian sample.

#### Work and Family Orientation scale.

The Work and Family Orientation Scale (WOFO) [48] measures different aspects of achievement motivation. The scale includes 19 items in three dimensions: Work Orientation (6 items), Mastery (8 items), and Competitiveness (5 items). Higher scores reflect higher levels of achievement motivation. We used a Korean translation of the scale developed through the same process as that used for COPS. The Cronbach’s alpha and McDonald’s omega values were.81/.81 for work,.72/.74 for mastery, and.79/.79 for competitiveness. In the Hungarian sample, these values were.72/.76,.68/.70, and.82/.82, respectively.

### Data analysis

Statistical analyses were performed using SPSS 29 and Mplus 8 [49]. Descriptive statistics and partial correlation coefficients were calculated using SPSS 29. The reliability coefficients (Cronbach’s alpha and McDonald’s omega), the structure and measurement invariance of the MCOI, and its criterion-related validity were tested using Mplus 8. The weighted least squares mean and variance adjusted (WLSMV) estimator was employed, as it produces less biased estimates than maximum likelihood for ordinal items [50]. Due to the ordinal nature of the items, Cronbach’s alpha and McDonald’s omega coefficients were computed based on the polychoric correlation matrix. Missing data were handled using Mplus’s default procedure for WLSMV estimation, which employs pairwise-present analysis. Missing responses were present only in the Hungarian sample, accounting for.65% of the data.

To evaluate measurement invariance, several confirmatory factor analysis (CFA) models were used to compare the MCOI’s factor structure between the Korean and Hungarian samples. Prior to these analyses, separate CFAs were conducted for each country to verify the adequacy of the modified four-factor model. Statistical constraints of increasing stringency were employed to either support or reject the comparability of items on the scales between the two groups. Configural invariance requires identical items with an equal number of factors across groups without the need for equivalent estimated parameters. Once configural invariance was confirmed, a scalar invariance model was directly tested by simultaneously constraining both factor loadings and thresholds across groups, following recommendations for the analysis of ordinal data. In such cases, it has been suggested that separate metric invariance testing may be bypassed, with loadings and thresholds constrained simultaneously, as both jointly influence item response probabilities [51,52]. Following the establishment of scalar invariance, factor variance invariance was tested first, and then factor covariance invariance was assessed to determine whether the correlation patterns among the four factors were invariant.

We assessed the goodness of fit using the following indices: chi-square, CFI, RMSEA with a 90% confidence interval, and SRMR. Acceptable fits were indicated by CFI values above.90, RMSEA less than.10, and SRMR values less than.08. Testing for invariance involved a sequence of nested model comparisons that progressively imposed more demanding constraints on the samples. For the chi-square difference test, we used Mplus’s DIFFTEST procedure, which is recommended for WLSMV estimation. As the chi-square test of invariance can be overly stringent, we also considered ΔCFI, ΔRMSEA, and ΔSRMR less than.01 as indicators of equivalent model fit [39].

After establishing measurement invariance, we examined criterion-related validity and compared the groups. For criterion measures that demonstrated measurement invariance, we conducted multigroup structural equation models regressing each measure on the MCOI factors. The analytical approach involved comparing a two-group model in which the paths from competitive orientations to a criterion factor were freely estimated, with another two-group model in which these paths were constrained to be invariant across samples. For measures that did not support measurement invariance between groups, we conducted partial correlation analyses within each group [53,54].

## Results

### Descriptive statistics and internal consistency

Descriptive statistics are presented in Table 1. Skewness and kurtosis values for all items and scale scores were below 3 and 8, respectively, indicating that the assumption of normality was met at both the item and scale levels. Including the revised MCOI, Cronbach’s alpha values were all above.68, and McDonald’s omega values were all above.70. Reliability coefficients above.70 are generally considered acceptable. As McDonald’s omega is regarded as a complementary index that may provide a less biased estimate compared to Cronbach’s alpha under certain conditions [55], these results suggest that all scales demonstrated adequate reliability.

**Table 1:** Descriptive statistics.

Scales	Range	Mean (*SD*)		Skewness		Kurtosis	
		KOR	HUN	KOR	HUN	KOR	HUN
MCOI SDCO	1-6	3.64 (1.04)	4.96 (.93)	−.10	−1.24	.06	1.66
MCOI HCO		2.99 (1.20)	2.50 (1.14)	.19	.70	−.52	−.02
MCOI ADCA		4.12 (.90)	2.24 (1.06)	−.15	1.06	−.01	.90
MCOI ADCA^a^		4.19 (1.07)	2.35 (1.13)	−.27	.97	−.34	.65
MCOI LIC		3.86 (.90)	2.62 (1.07)	−.10	.72	−.01	.52
MCOI LIC^a^		3.68 (1.19)	2.54 (1.12)	−.14	.74	−.37	.53
CD-RISC	1-5	3.27 (.67)	3.82 (.59)	−.18	−.30	.35	.27
COPS positive		3.26 (.78)	4.21 (.56)	−.26	−.48	.45	.10
COPS negative		3.02 (.82)	1.85 (.72)	−.21	.90	−.29	.99
WOFO competition		3.10 (.76)	3.49 (.76)	−.13	−.22	.31	−.02
WOFO mastery		2.97 (.61)	3.50 (.45)	−.36	.16	.88	.29
WOFO work		3.74 (.63)	4.25 (.46)	−.26	−.45	.33	−.20
Positivity		3.03 (.74)	3.70 (.61)	.10	−.29	.01	−.07
APS-R high standards		3.29 (.79)	4.07 (.67)	−.11	−.61	.07	−.21
APS-R discrepancy		3.03 (.77)	2.61 (.91)	−.03	.33	−.08	−.54

*SD* = standard deviation; MCOI = Multidimensional Competitive Orientation Inventory; SDCO = self-developmental competitive orientation; HCO = hypercompetitive orientation; ADCA = anxiety-driven competition avoidance; LIC = lack of interest in competition; CD-RISC = Connor-Davidson Resilience Scale; COPS = Consequences of Perfectionism; WOFO = Work and Family Orientation; APS-R = Almost Perfect Scale-Revised; KOR = South Korea; HUN = Hungary. ^a^ One item from the subscale was removed.

### Separate sample measurement models

Before testing the measurement invariance between the Korean and Hungarian samples, we tested the original four-factor model (verified in Hungarian samples in [17]) for the Korean samples using CFA. The fit indices for the Korean sample and four-factor MCOI model were: *χ*^*2*^(48, *N* = 350) = 362.492, *p* < .001, CFI = .90, RMSEA = .14, 90% CI [.124,.150], SRMR = .06. The standardized factor loadings ranged from.08 to.98. Some fit indices did not meet the criteria, and the factor loadings of a few items were significantly low. Items should have a factor loading of at least.4 to be deemed important [56]. Consequently, two items with factor loadings of.08 and.10, respectively, were eliminated: “There is always something I’d rather do than taking part in a competitive situation” (lack of interest), and “Even the smallest competition makes me feel anxious” (anxiety-driven competition avoidance).

After removing the two low-loading items, the fit indices of the CFA in the Korean sample were highly acceptable: *χ*^*2*^(29, *N* = 350) = 76.700, *p* < .001, CFI = .98, RMSEA = .07, 90% CI [.050,.087], SRMR = .03. The standardized factor loadings ranged from.51 to.94. To check if the modified version is equivalent across subgroups in the Korean sample, we conducted measurement invariance tests across subgroups (i.e., age and sex). As shown in Table 2, both configural and scalar invariance were supported for age (underage vs. adult) and gender (male vs. female) groups, confirming the equivalence of the measurement model across these subpopulations.

**Table 2:** Measurement invariance models on the MCOI and criterion factors.

Model	χ^2^	df	Δχ^2^	Δ df	*p*	RMSEA (90% CI)	Δ RMSEA	CFI	Δ CFI	SRMR	Δ SRMR
*MCOI – Within Korean sample (gender)*											
Configural	185.064	94				.074 (.058,.090)		.966		.037	
Scalar	180.947	100	4.531	6	.605	.068 (.052,.084)	.006	.970	.004	.038	.001
*MCOI – Within Korean sample (age)*											
Configural	161.467	94				.064 (.047,.081)		.978		.038	
Scalar	155.031	100	4.557	6	.602	.056 (.038,.073)	.008	.982	.004	.039	.001
*MCOI – Between South Korean and Hungarian Samples*											
Configural	376.117	94				.093 (.083,.103)		.958		.035	
Scalar	386.340	100	23.288	6	.000	.091 (.081,.101)	.002	.957	.001	.037	.002
Factor variance	379.090	104	23.771	4	.000	.087 (.078,.097)	.004	.959	.002	.039	.002
Factor covariance	419.964	110	49.916	6	.000	.090 (.081,.099)	.003	.954	.005	.054	.015
*CD-RISC – Between South Korean and Hungarian Samples*											
Configural	430.194	98				.099 (.089,.109)		.923		.046	
Scalar	410.687	107	15.899	9	.000	.091 (.081,.100)	.008	.930	.007	.047	.001
*COPS – Between South Korean and Hungarian Samples*											
Configural	210.563	96				.059 (.048,.069)		.981		.035	
Scalar	254.339	104	46.208	8	.000	.065 (.055,.075)	.006	.975	.006	.036	.001

MCOI = Multidimensional Competitive Orientation Inventory; CD-RISC = Connor-Davidson Resilience Scale; COPS = Consequences of Perfectionism; df = degrees of freedom; RMSEA = Root Mean Square Error of Approximation; ΔRMSEA = Absolute Change in Root Mean Square Error of Approximation; CFI = Comparative Fit Index; ΔCFI = Absolute Change in Comparative Fit Index; SRMR = Standardized Root Mean Square Residual; Δ SRMR = Absolute Change in Standardized Root Mean Square Residual; Chi-square difference tests between nested models were conducted using DIFFTEST option in Mplus.

Lastly, prior to conducting detailed measurement invariance tests, we conducted CFA on the Hungarian sample after eliminating the same two items: *χ*^*2*^(29, *N* = 343) = 87.259, *p* < .001, CFI = .99, RMSEA = .08, 90% CI [0.058, 0.095], SRMR = .03. Standardized factor loadings ranged from.78 to.92. As the fit indices and factor loadings were acceptable, subsequent analyses (configural and scalar invariance tests) were conducted between the two countries using the revised version of MCOI. The detailed CFA results for the Korean and Hungarian samples are presented in Supporting Information S2 Table.

### Measurement invariance

The goodness-of-fit results for the measurement invariance models are summarized in Table 2. Table 3 displays the factor loadings estimated freely under the configural invariance model. We conducted a configural invariance test by examining a baseline model with no parameters constrained across the two countries. The configural model showed acceptable fit, as all fit indices (RMSEA, CFI, and SRMR) met the criteria for adequacy. Only the upper bound of the RMSEA 90% CI marginally exceeded.10 (upper = .103). Therefore, we tested a scalar model by simultaneously constraining both factor loadings and item thresholds across groups. The absolute changes in fit indices between the configural and scalar models met recommended thresholds (ΔCFI, ΔRMSEA, and ΔSRMR < .01) [39], indicating support for configural and scalar invariance of the MCOI across the Korean and Hungarian samples.

**Table 3:** Estimates based on the configural invariance model.

Factor	South Korea			Hungary		
	*B*	*SE*	β	*B*	*SE*	β
*SDCO*						
Item 1	1.00	.00	.51	1.00	.00	.76
Item 2	2.39	.33	.82	1.67	.17	.90
Item 3	2.49	.38	.83	1.69	.19	.86
*HCO*						
Item 4	1.00	.00	.87	1.10	.00	.91
Item 5	.88	.13	.84	.69	.09	.79
Item 6	.75	.10	.79	.64	.08	.80
*ADCA*						
Item 7	1.00	.00	.94	1.00	.00	.96
Item 8	.26	.12	.58	.32	.12	.79
*LIC*						
Item 9	1.00	.00	.81	1.00	.00	.89
Item 10	.79	.12	.73	.65	.07	.76

*Note.* B = Unstandardized factor loadings; β = Standardized factor loadings; SDCO = self-developmental competitive orientation; HCO = hypercompetitive orientation; ADCA = anxiety-driven competition avoidance; LIC = lack of interest in competition; KOR = South Korea; HUN = Hungary; For unstandardized coefficients, the loading of the first indicator of each factor was fixed at 1.00; All factor loadings are significant, *p* < .001.

### Structural invariance

With scalar invariance established, we next examined structural invariance. We first assessed invariance of factor variances while retaining the scalar constraints; as summarized in Table 2, factor variance invariance was supported. We then evaluated equality of factor covariances by comparing the factor variance invariance model, in which covariances were freely estimated across groups, to a model in which all factor covariances were constrained to be equal. The correlations in Table 4 were obtained from the factor variance invariance baseline model. Constraining all covariances resulted in significantly worse model fit according to the DIFFTEST, with an SRMR change as shown in Table 2.

**Table 4:** Freely estimated standardized factor correlations of MCOI.

	SDCO		HCO		ADCA		LIC	
	KOR	HUN	KOR	HUN	KOR	HUN	KOR	HUN
SDCO	–							
HCO	.62	.43	–					
ADCA	−.45	−.84	−.24	−.30	–			
LIC	−.52	−.79	−.28	−.34	.73	.71	–	

SDCO = self-developmental competitive orientation; HCO = hypercompetitive orientation; ADCA = anxiety-driven competition avoidance; LIC = lack of interest in competition factor; KOR = South Korea; HUN = Hungary. All correlations are significant, *p* < .001.

Accordingly, we conducted further analyses to identify where these differences emerged. We compared the factor variance invariance model with a model that constrained only a single covariance to be equal across groups. The correlation between self-developmental competitive orientation and anxiety-driven competition avoidance showed the most notable difference, as supported by both the DIFFTEST and changes in fit indices: Δ*χ*^*2*^(1, *N* = 693) = 36.693, *p* < .001, ΔCFI = .010, ΔRMSEA = .010. The negative correlation between these two orientations was stronger in the Hungarian sample. According to DIFFTEST, the positive correlation between self-developmental competitive orientation and hypercompetitive orientation was stronger in the Korean sample, while the negative correlation between self-developmental competitive orientation and lack of interest was stronger in the Hungarian sample: Δ*χ*^*2*^(1, *N* = 693) = 7.471, *p* < .01, Δ*χ*^*2*^(1, N = 693) = 14.573, *p* < .001, respectively.

### Criterion-related validity

To assess criterion-related validity, the measurement invariance of the five measures used by Orosz et al. [17] was tested between the Korean and Hungarian samples. The results for the measures that satisfied measurement invariance are summarized in Table 2. The scalar invariance of resilience and COPS was supported. Therefore, these two variables were used to compare criterion-related validity between the two countries. For the analysis, two models were compared: one with freely estimated paths from competitive orientations to the criterion factor and the other with paths that were constrained to be equal across samples. Table 5 presents the regression estimates.

**Table 5:** Criterion-related validity: regression estimates.

	Korea			Hungary		
	*B*	*SE*	β	*B*	*SE*	β
*CD-RISC*						
SDCO	.79	.10	.76^c^	.52	.12	.67^c^
HCO	−.17	.05	−.25^b^	−.09	.04	−.14^a^
ADCA	−.54	.18	−.40^b^	−.25	.11	−.27^a^
LIC	.53	.18	.36^b^	.48	.14	.36^c^
*COPS positive*						
SDCO	.61	.11	.48^c^	.89	.14	.95^c^
HCO	−.01	.06	−.01	−.09	.06	−.11
ADCA	.12	.19	.07	.27	.12	.24^a^
LIC	−.21	.22	−.12	.35	.18	.21
*COPS negative*						
SDCO	.13	.12	.10	−.19	.15	−.17
HCO	.03	.07	.04	.28	.07	.30^c^
ADCA	.46	.21	.28^a^	.57	.14	.43^c^
LIC	.13	.22	.07	−.41	.21	−.22^a^

^a^*p* < .05. ^b^*p* < .01. ^c^*p* < .001. *B* = Unstandardized beta, *SE* = Standard error, β = Standardized beta, CD-RISC = Connor-Davidson Resilience Scale, COPS = Consequences of Perfectionism.

For resilience, although the DIFFTEST indicated a statistically significant decrement in fit when constraining all paths to be equal, changes in global fit indices were negligible: Δ*χ*^*2*^(4, *N* = 693) = 39.540, *p* < .001, ΔCFI = .005, ΔRMSEA = .003, ΔSRMR = .002. In both samples, self-developmental competitive orientation and lack of interest were positively associated with resilience, while anxiety-driven competition avoidance and hypercompetitive orientation were negatively associated with resilience.

Similarly for COPS, only the DIFFTEST indicated a significant difference in the overall model comparison: Δ*χ*^*2*^(8, *N* = 693) = 25.726, *p* < .001, ΔCFI = .001, ΔRMSEA = .001, ΔSRMR = .006. For the positive consequences of perfectionism, self-developmental competitive orientation was significantly and positively associated in both samples, whereas anxiety-driven competition avoidance showed a significant positive association only in the Hungarian sample. For the negative consequences of perfectionism, anxiety-driven competition avoidance was significantly and positively associated in both samples; only in the Hungarian sample, self-developmental competitive orientation and lack of interest were significantly and negatively associated, while hypercompetitive orientation was significantly and positively associated.

Results from both regression analyses and factor invariance analyses suggested the possibility of cultural differences. Thus, for the three scales (APS-R, POS, WOFO) for which measurement invariance was not established, we conducted separate partial correlation analyses within each country to further confirm additional criterion-related validity. When calculating the partial correlations between competitive factors and outcomes, we controlled for the other competitive factors. The results of each sample’s correlations between the different competitive orientations and adaptive-maladaptive variables are presented in Table 6.

**Table 6:** Partial Correlations and Correlations between Korean and Hungarian Samples on MCOI.

	SDCO		HCO		ADCA		LIC		WOFO competition		WOFO mastery		WOFO work		Positivity		APS-R high standards	
	KO	HU	KO	HU	KO	HU	KO	HU	KO	HU	KO	HU	KO	HU	KO	HU	KO	HU
WOFO competition	.26^c^	.20^c^	.38^c^	.36^c^	.10	.07	−.30^b^	−.35^c^	–									
WOFO mastery	.33^c^	.28^c^	.12^a^	−.04	−.21^c^	.03	.05	.00	.49^c^	.26^c^	**–**							
WOFO work	.30^c^	.19^b^	−.29^c^	−.02	.11	.04	−.06	−.02	.11^a^	.19^b^	.41^c^	.45^c^	–					
Positivity	.30^c^	.18^b^	−.03	−.03	−.20^a^	−.05	.08	.02	.33^c^	.06	.56^c^	.21^c^	.42^c^	.18^b^	–			
APS high standards	.28^c^	.17^b^	.13^a^	.11	.10	.19^b^	−.18^b^	−.19^b^	.50^c^	.43^c^	.45^c^	.43^c^	.20^c^	.39^c^	.33^c^	.07	–	
APS discrepancy	.03	.03	.18^b^	.15^b^	.16^b^	.22^c^	.15^b^	−.02	.11^a^	.15^b^	.12^a^	.06	−.14^b^	.03	−.28^c^	−.44^c^	.27^c^	.37^c^

^a^*p* < .05. ^b^*p* < .01. ^c^*p* < .001. SDCO = self-developmental competitive orientation; HCO = hypercompetitive orientation; ADCA = anxiety-driven competition avoidance; LIC = lack of interest in competition; CD-RISC = Connor-Davidson Resilience Scale; COPS = Consequences of Perfectionism; WOFO = Work and Family Orientation; APS-R = Almost Perfect Scale-Revised; KO = South Korea; HU = Hungary, To analyze the Hungarian sample under the same item conditions as the Korean sample, four items were removed from the APS-R before conducting the analysis.

In both the Hungarian and Korean samples, the overall pattern of associations between competitive orientations and other variables was consistent with expectations. Effect sizes were interpreted in accordance with Cohen’s [57] guidelines (.10 = small,.30 = medium,.50 = large). Self-developmental competitive orientation was positively associated with achievement motivation including competitiveness (*r*_*Kor*_ = .26, *p* < .001, small; *r*_*Hun*_ = .20, *p* < .001, small), mastery (*r*_*Kor*_ = .33, *p* < .001, medium; *r*_*Hun*_ = .28, *p* < .001, small), and work (*r*_*Kor*_ = .30, *p* < .001, medium; *r*_*Hun*_ = .19, *p* < .01, small). This orientation was also associated with adaptive outcomes such as positivity in both countries (*r*_*Kor*_ = .30, *p* < .001, medium; *r*_*Hun*_ = .18, *p* < .01, small).

Hypercompetitive orientation (*r*_*Kor*_ = .18, *p* < .01, small; *r*_*Hun*_ = .15, *p* < .01, small) and anxiety-driven competition avoidance (*r*_*Kor*_ = .16, *p* < .01, small; *r*_*Hun*_ = .22, *p* < .001, small) were linked to maladaptive outcomes such as discrepancy. However, only hypercompetitive orientation was related to motivation to compete (*r*_*Kor*_ = .38, *p* < .001, medium; *r*_*Hun*_ = .36, *p* < .001, medium).

Lack of interest was negatively associated with both competition (*r*_*Kor*_ = −.30, *p* < .01, medium; *r*_*Hun*_ = −.35, *p* < .001, medium) and high standards (*r*_*Kor*_ = −.18, p < .01, small; *r*_*Hun*_ = −.19, *p* < .01, small) in both countries; however, it was positively associated with discrepancy only in South Korea (*r*_*Korea*_ = .15, *p* < .01, small). Barring lack of interest, the overall pattern of adaptiveness of each orientation was largely consistent across countries, with some differences in detail (see Tables 5 and 6).

## Discussion

To cross-culturally validate the MCOI and understand the cultural differences in the relationships between the four competitive orientations and related psychological characteristics, this study compared South Korean and Hungarian data to answer two research questions.

### Is the factor structure of MCOI invariant across South Korean and Hungarian samples?

The results suggest invariance in the factor structure between Hungarians and Koreans. However, it is important to note that this result was obtained by removing two items, which calls for caution when employing the scale across the two countries. The two items inherently convey a strong aversion to engaging in competition, but in the context of the highly competitive societal atmosphere in Korea, this may not have resonated effectively. Regarding measurement invariance, configural and scalar invariances were observed. The same groupings of items explained competitive orientation as an invariant, culturally universal construct. These results are consistent with previous studies showing that the MCOI maintained its factor structure in both Western (e.g., France; [58]) and non-Western (e.g., China; [27]) contexts. Regarding the inter-factor relationships, correlations between the factors showed that two factors related to approaching motivation (hypercompetitive and self-developmental competitive orientations) were positively correlated. Furthermore, the remaining factors related to distancing motivation (avoidance and lack of interest) were also positively correlated. A clear negative relationship was observed between the two opposing motivations. Overall, evidence for configural and scalar invariance, along with the consistency in the directions of inter-factor relationships across groups, supports the MCOI as a psychometrically stable measure of adaptive, maladaptive, and neutral competitive orientations in both South Korea and Hungary.

Meanwhile, although the magnitude of these differences was small, a statistically significant difference was observed in the strength of the correlations between the factors. Comparing South Koreans with Hungarians, stronger correlations were observed between the two orientations reflecting approaching motivation. However, the negative correlations between the self-developmental competitive orientation and the two orientations of distancing motivation of South Koreans were weaker than those for Hungarians. That is, South Koreans may have perceived the competitive orientations as less distinct from one another than the Hungarians did. In South Korea’s highly competitive yet collectivistic culture, individuals face contradictory pressures to excel and achieve outstanding results [31] while avoiding standing out or boasting [26]. These opposing demands may foster the coexistence of approaching and distancing competitive orientations. These results are consistent with Fülöp’s [20,21] proposal that individualistic-collectivistic cultural differences can affect members’ perceptions of and attitudes toward competition. Similar to Japanese participants in Fülöp’s studies [20,21], who integrated competition with cooperation, collectivistic South Koreans exhibited mixed competitive orientations (e.g., self-developmental but anxiety-driven, depending on the situation) compared with individualistic Hungarians.

Cultural differences in self-concept may also help interpret these results. Collectivistic South Korean culture fosters an interdependent self-concept, allowing individuals to flexibly adopt approaching or distancing motivations toward competition depending on situational demands [59]. For instance, even those with high anxiety may be socially encouraged to compete in contexts such as college entrance and job promotion. In contrast, individualistic Hungarian culture promotes an independent self-concept that remains relatively stable despite external demands [59]. Because people with an independent self-concept tend to minimize contradictions [60], Hungarians may be more inclined than Koreans to maintain a consistent orientation across competitive situations.

### Do the relationships between competitive orientations and psychological characteristics differ between South Korean and Hungarian cultures?

We observed patterns that were largely consistent across cultures in the psychological characteristics associated with competitive orientations. Although cross-national differences were generally small and some variables required further examination after establishing measurement invariance, some specific cross-national differences emerged.

First, self-developmental competitive orientation is considered the most adaptive type [12,17]. Here, participants (South Koreans and Hungarians) with this orientation were prone to putting effort into work, and had high levels of both achievement motivation and willingness to take on challenging tasks. They also had high levels of positivity, consisting of optimism, life satisfaction, and self-esteem, as well as resilience. These findings are consistent with prior findings on the positive and adaptive aspects of competition [19,61]. This orientation showed consistent associations with all criterion-related variables across cultural groups, indicating similar characteristics regardless of culture.

For hypercompetitive orientation, both South Koreans and Hungarians exhibited a strong desire to outperform others and showed psychologically negative signs, such as a perceived discrepancy between the ideal and actual selves. These findings align with previous research linking an excessive preoccupation with winning to psychological maladjustment [11,14]. Notably, South Koreans were willing to take on challenges but reported less motivation to sustain personal efforts over time, as reflected in the WOFO mastery and work subscales. This pattern may reflect the influence of a collectivistic cultural context, in which external expectations to achieve are high, but intrinsic, self-directed motivation may be undermined by the pressure to conform to social norms [26].

Anxiety-driven competition avoidance was also universally maladaptive. As Ryckman et al. [62] found, participants with anxiety-driven competition avoidance showed low resilience, along with high self-discrepancy. These results support prior research suggesting that an avoidant attitude toward competition due to anxiety can be maladaptive [13,19]. South Koreans with this orientation tended to avoid challenging tasks and showed lower levels of positivity, including self-esteem, whereas Hungarians in this group held higher personal standards. Although the specific associated variables differed between the two countries, in both cases the pressure to engage in high-achievement tasks was associated with the motivation to avoid competition.

In contrast to the other three orientations, lack of interest showed cultural differences in terms of adaptiveness. Although lack of interest was universally related to low motivation for competition and to relatively higher resilience than that among those who anxiously avoided competition, in South Koreans, lack of interest was positively correlated with the discrepancy between the ideal and actual selves. This self-discrepancy has been understood as a source of stress and shame [63]. There may be two tentative interpretations of these results. First, the collectivistic cultural trait of saving face and the resulting emotional ambivalence may have influenced this association. Displaying a lack of interest may serve as a strategy to conceal anxiety about participating in and/or losing a competition. This ambivalence interferes with emotional clarity, which is considered an important aspect of effective emotion regulation [64,65]. While this interpretation is provisional, it highlights the need to closely examine the factors related to a lack of interest when studying samples from collectivistic cultures.

Second, South Korean participants who scored high on lack of interest may have genuinely lacked interest in winning; however, the competitive social atmosphere in South Korea could have caused them to experience stress about not conforming to social norms. Since an interdependent self-concept that develops in collectivistic cultures leads individuals to take social expectations and others’ opinions into account when evaluating their own abilities and self-worth [66,67], a lack of interest in such competitive social environments can raise a sense of inadequacy and self-discrepancy as individuals internalize these expectations. A high level of self-discrepancy may function as both a source (anxiety and emotional ambivalence) and a consequence (feeling inadequate in the competitive South Korean social atmosphere) of lack of interest in competition.

### Implications, limitations, and future directions

This study provides valuable insights for the literature and practice of psychological treatment for people who have difficulties with competition. First, it cross-culturally validated the MCOI using Korean and Hungarian samples and supported its use across the two cultures. Thus, more reliable studies can be conducted using the MCOI outside Western countries. Second, this study provided empirical evidence of cultural differences between individualistic and collectivistic cultures beyond the inferences made in prior studies, such as Fülöp [20,21]. Finally, the relationships between different competitive orientations and psychological characteristics could help practitioners understand their clients’ struggles. Young clients, such as high school students, may experience significant stress about academic competitions, and the findings of this study could provide a cue for counselors to consider culture and cultural traits such as emotional ambivalence when conceptualizing their clients’ circumstances.

Despite these contributions, this study has several limitations. First, administering the MCOI solely as a self-report measure without supplementing it with other questionnaires or direct observation by researchers or practitioners means that important information may be missing. Future studies should interpret MCOI results alongside interpersonal behaviors and outcomes. Second, specific cultural traits may differ across collectivistic countries. Therefore, the results of this study should be interpreted with caution. Researchers should also compare more than two collectivistic countries to determine whether other specific cultural traits contribute to the endorsement, use, or expression of competitive orientations. Finally, longitudinal studies are required to investigate whether competitive orientations change over time or remain stable (i.e., trait-like). Studying competitive orientations over time or across situational changes (e.g., graduation, changing jobs/fields, or moving to another area) may help researchers and practitioners better understand the nature of competitive orientations, including whether they are closely related to personal traits or are context-laden.

## Supporting information

S1 Data Raw data set from the South Korean sample. (CSV)

S2 Table The detailed CFA results for the Korean and Hungarian samples. (DOCX)
