# Supplementary material for: Cross-cultural measurement invariance of the multidimensional competitive orientation inventory: Non-clinical samples in South Korea and Hungary
Source: PLoS One. 2025 Dec 26;20(12):e0337685. doi: 10.1371/journal.pone.0337685 (PMC12742798; doi:10.1371/journal.pone.0337685)
Supplement: S2 Table — (DOCX) [file pone.0337685.s002.docx]

**Table S2. CFA estimates from separate analyses for South Korea and Hungary**

| **Factor** | | **South Korea** | | | | | | | | | | **Hungary** | | | | | | | | |
| --- | --- | --- | --- | --- | --- | --- | --- | --- | --- | --- | --- | --- | --- | --- | --- | --- | --- | --- | --- | --- |
|  |  | ***B*** | | | ***SE*** | | | **β** | | | | ***B*** | | | ***SE*** | | | **β** | | |
| *SDCO* | |  | |  | | |  | | |  | |  | |  | | |  | | |  |
| Item 1 | | 1.00 | | | .00 | | | .51 | | | | 1.00 | | | .00 | | | .80 | | |
| Item 2 | | 1.61 | | | .13 | | | .82 | | | | 1.08 | | | .04 | | | .87 | | |
| Item 3 | | 1.63 | | | .14 | | | .83 | | | | 1.06 | | | .05 | | | .85 | | |
| *HCA* | |  | |  | | |  | | |  | |  | |  | | |  | | |  |
| Item 4 | | 1. 00 | | | .00 | | | .87 | | | | 1.10 | | | .00 | | | .89 | | |
| Item 5 | | .96 | | | .04 | | | .84 | | | | .88 | | | .04 | | | .78 | | |
| Item 6 | | .91 | | | .04 | | | .79 | | | | .94 | | | .04 | | | .83 | | |
| *ADCA* | |  | |  | | |  | | |  | |  | |  | | |  | | |  |
| Item 7 | | 1.00 | | | .00 | | | .94 | | | | 1.00 | | | .00 | | | .92 | | |
| Item 8 | | .62 | | | .06 | | | .58 | | | | .90 | | | .04 | | | .83 | | |
| *LIC* | |  | |  | | |  | | |  | |  | |  | | |  | | |  |
| Item 9 | | 1.00 | | | .00 | | | .81 | | | | 1.00 | | | .00 | | | .86 | | |
| Item 10 | | .91 | | | .05 | | | .73 | | | | .92 | | | .05 | | | .79 | | |
|  | **SDCO** | | | | | **HCA** | | | | | **ADCA** | | | | | **LIC** | | | | |
|  | KOR | | HUN | | | KOR | | | HUN | | KOR | | HUN | | | KOR | | | HUN | |
| SDCA | - | | | | |  | | |  | |  | |  | | |  | | |  | |
| HCA | .64 | | .42 | | | - | | | | |  | |  | | |  | | |  | |
| ADCA | -.50 | | -.80 | | | -.25 | | | -.29 | | - | | | | |  | | |  | |
| LIC | -.55 | | -.77 | | | -.28 | | | -.33 | | .79 | | .69 | | | - | | | | |

*Note.* B = Unstandardized factor loadings; β = Standardized factor loadings; SDCO = self-developmental competitive orientation; HCO = hypercompetitive orientation; ADCA = anxiety-driven competition avoidance; LIC = lack of interest in competition; KOR = South Korea; HUN = Hungary; For unstandardized coefficients, the loading of the first indicator of each factor was fixed at 1.00; All factor loadings and correlations are significant, *p* < .001.
